# Why drug exposure is frequently associated with T-cell mediated cutaneous hypersensitivity reactions

**DOI:** 10.3389/ftox.2023.1268107

**Published:** 2023-09-19

**Authors:** James Line, Eleanor Saville, Xiaoli Meng, Dean Naisbitt

**Affiliations:** Department of Pharmacology and Therapeutics, University of Liverpool, Liverpool, United Kingdom

**Keywords:** T-cell, skin, drug hypersensitivity, immunotoxicology, drug metabolism, memory responses

## Abstract

Cutaneous hypersensitivity reactions represent the most common manifestation of drug allergy seen in the clinic, with 25% of all adverse drug reactions appearing in the skin. The severity of cutaneous eruptions can vastly differ depending on the cellular mechanisms involved from a minor, self-resolving maculopapular rash to major, life-threatening pathologies such as the T-cell mediated bullous eruptions, i.e., Stevens Johnson syndrome/toxic epidermal necrolysis. It remains a significant question as to why these reactions are so frequently associated with the skin and what factors polarise these reactions towards more serious disease states. The barrier function which the skin performs means it is constantly subject to a barrage of danger signals, creating an environment that favors elicitation. Therefore, a critical question is what drives the expansion of cutaneous lymphocyte antigen positive, skin homing, T-cell sub-populations in draining lymph nodes. One answer could be the heterologous immunity hypothesis whereby tissue resident memory T-cells that express T-cell receptors (TCRs) for pathogen derived antigens cross-react with drug antigen. A significant amount of research has been conducted on skin immunity in the context of contact allergy and the role of tissue specific antigen presenting cells in presenting drug antigen to T-cells, but it is unclear how this relates to epitopes derived from circulation. Studies have shown that the skin is a metabolically active organ, capable of generating reactive drug metabolites. However, we know that drug antigens are displayed systemically so what factors permit tolerance in one part of the body, but reactivity in the skin. Most adverse drug reactions are mild, and skin eruptions tend to be visible to the patient, whereas minor organ injury such as transient transaminase elevation is often not apparent. Systemic hypersensitivity reactions tend to have early cutaneous manifestations, the progression of which is halted by early diagnosis and treatment. It is apparent that the preference for cutaneous involvement of drug hypersensitivity reactions is multi-faceted, therefore this review aims to abridge the findings from literature on the current state of the field and provide insight into the cellular and metabolic mechanisms which may contribute to severe cutaneous adverse reactions.

## 1 Introduction

Adverse drug reactions (ADRs) occur when therapeutically relevant doses of drugs result in harmful and unintended responses. ADRs can be divided into two major categories: 1) Type A reactions, which represent 85%–90% of ADR cases and can be predicted based on the known pharmacology of the drug where adverse effects are the result of exaggerated drug action or off-target interactions and 2) Type B reactions, which make up the remaining 10%–15% of ADRs (Table 1). These are idiosyncratic and unpredictable and often have an innate and adaptive immune basis, with T-cells being commonly implicated (Waller, 2011).

**TABLE 1:** A brief overview of drug hypersensitivity reaction types and mechanisms.

Type	Description	Prognosis	Mediator	Mechanism
A	Predictable	High morbidity	Exaggerated pharmacology and off-target action	
		Low mortality		
B	Unpredictable		I. IgE	Mast cell activation leading to histamine release
		Low morbidity	II. IgG/IgM	Cytotoxic response mediated by complement
		High mortality	III. IgG/IgM	Neutrophil and complement activation
			IV. T-cells	Effector T-cell and macrophage activation

A 2021 meta-analysis suggested that around 8% of primary care patients experience ADRs, a number which inflates to 10%–20% in hospitalised patients demonstrating the huge burden of ADRs on healthcare settings (Pirmohamed et al., 2004; Insani et al., 2021). Analysis conducted in 2022 in one of NHS England’s Medical Trusts found a prevalence of 16.5% of total admissions being directly related to ADRs with extrapolation revealing a national estimated annual cost of £2.21 billion (Osanlou et al., 2022). Cutaneous reactions make up a quarter of adverse drug responses seen in the clinic and therefore understanding the underlying mechanisms of such responses is vital in reducing both risk to patient safety and the financial burden placed on the health service.

However, an immediate obstacle in elucidating the mechanisms of cutaneous ADRs is the diversity of both clinical manifestations and the range of immune mediators present (Table 1). The matter is further complicated by the uncoordinated and generalised nature of drug hypersensitivity reactions (DHRs) when compared to the more coordinated and localised immune response to pathogens (Bircher, 2022).

Antibody mediated type I-III hypersensitivity reactions occur rapidly upon drug administration where IgE release triggers the mast cell degranulation and histamine release often associated with anaphylactic reactions. The IgG and IgM mediated type II and III reactions typically involve cytotoxic activity and complement activation resulting in conditions such as haemolytic anaemia and serum sickness (Justiz Vaillant et al., 2023). Contrastingly type IV reactions, also known as delayed-type hypersensitivity reactions, are mediated by drug reactive T-cells and these reactions will be the focus of this review.

### 1.1 Cutaneous reactions

ADRs most commonly target the skin, with several diverse reactions having been observed and characterised. Most cutaneous hypersensitivity cases present as maculopapular exanthema (MPE)—a minor skin rash consisting of lesions which cover the face, limbs, and torso (Bircher, 2022; Khandpur and Ahuja, 2022). A small proportion of patients however may experience severe cutaneous adverse reactions (SCARs) including acute generalised exanthematous pustulosis (AGEP), drug reaction with eosinophilia and systemic symptoms (DRESS), or Stevens-Johnson syndrome/toxic epidermal necrolysis (SJS/TEN). Although these reactions vary in terms of effector function, there exists overlap where milder reactions such as MPE can be the initial presentation of more serious ADRs such as SJS/TEN.

#### 1.1.1 Maculopapular exanthema

MPE is the most common cutaneous ADR, which typically presents between 1 and 2 weeks following initial drug exposure. Symptoms consist of flat macule and raised papule lesions of 1–5 mm in diameter with minimal systemic upset (Pinto Gouveia et al., 2016). The macules and papules initially appear on the torso before spreading bilaterally to the limbs and often merging into plaques. MPE can also present alongside other cutaneous ADR presentations such as urticaria, also known as hives, and purpura, a purple rash caused by damage to small blood vessels (Ernst and Giubellino, 2022). MPE is considered to be a mild cutaneous reaction which typically self-resolves in around 7–14 days following drug withdrawal (Crisafulli et al., 2019). Re-appearance of symptoms following drug re-challenge is considered the gold-standard, if not an essential criterion, for the diagnosis of drug-induced MPE. However most patients do not consent to re-exposure and the time-consuming procedures involved (Singh et al., 2017).

The pathogenesis of MPE is not fully characterised and the polymorphic nature of immune responses induce considerable variation between patients and drugs. It is generally agreed upon that drugs are presented to antigen-specific CD4^+^ and CD8^+^ T-cells leading to inflammatory cytokine production, skin infiltration and subsequent cytotoxicity (Ernst and Giubellino, 2022). Several studies have identified CD4^+^ T-cells as the main effectors in MPE. Indeed, it is suggested that CD4^+^ T-cell activation results in maculopapular reactions, where both raised and flat eruptions are seen, whereas CD8^+^ T-cell activation is more commonly associated with bullous skin reactions like SJS/TEN (Hari et al., 2001; Fernandez et al., 2010). Antibiotic drugs such as the ß-lactams and sulfamethoxazole are commonly implicated in the development of drug-induced MPE.

#### 1.1.2 Fixed drug eruption

Fixed drug eruption (FDE) is a rarer form of type IV cutaneous DHR. Instead of widespread clinical manifestation, erythematous eruptions, or plaques of up to 10 cm in diameter occur in small, localised patches, and develop in the same location following each successive drug exposure. Antibiotics, especially co-trimoxazole, NSAIDs, and anti-convulsants are seen to be the most common culprit drugs (Shaker et al., 2022).

Whilst this pathology is poorly recognised and understood, in part by frequent misdiagnosis due to the similarities to conditions such as urticaria, it is thought to be mediated by resident CD8^+^ T-cells which secrete inflammatory mediators such as IFN-γ leading to local epidermal injury. Inflammation and damage are resolved following drug withdrawal. Hyperpigmentation is a long term sequela seen with FDE, where the macrophages responsible for phagocytosis of leaked melanin are retained within the skin (McClatchy et al., 2022). Memory CD8^+^ T-cells are also formed and remain in the basal layer of the epidermis at high concentrations for prolonged periods of time allowing swift re-activation in the same location upon drug re-exposure.

#### 1.1.3 Acute generalised exanthematous pustulosis

AGEP typically occurs within 48 h of drug ingestion and consists largely of non-follicular sterile pustules which self-resolve within 2 weeks following drug withdrawal (Feldmeyer et al., 2016). Presentation initially occurs in the main folds of skin before rapid spread to the rest of the torso and limbs in a matter of hours.

The pathogenesis of AGEP is associated with the action of drug-specific CD4^+^ and CD8^+^ T-cells which subsequently recruit neutrophils through secretion of IL-8. Neutropenia is observed in a significant proportion of AGEP patients, with *in vitro* analysis of patient derived T-cells reporting increased production of IL-8, the major chemokine responsible for neutrophil chemotaxis and activation (Britschgi et al., 2001; Feldmeyer et al., 2016; Metzemaekers et al., 2020). Immune cells local to the site of tissue injury further augment the development of AGEP with Langerhans cells presenting antigens to CD4^+^ T-cells and keratinocytes acting as antigen presenting cells to cytotoxic CD8^+^ T-cells (De et al., 2018a). The pencillin and sulphonamide families are commonly implicated causative agents alongside anti-fungals and anti-inflammatories.

#### 1.1.4 Drug reaction with eosinophilia and systemic symptoms

DRESS is a multi-organ condition which involves pustular and blistering skin eruptions, as well as haematological abnormalities and visceral organ involvement. DRESS patients often experience fever alongside pruritic exanthema with varying degrees of severity. Haematological abnormalities such as eosinophilia are also considered hallmarks of DRESS, with the liver and kidneys being the most commonly affected organs (Del Pozzo-Magaña and Liy-Wong, 2022). Viremia is a common clinical characteristic of DRESS, with up to three-quarters of patients experiencing viral reactivation of Human Herpes Virus, Epstein Barr Virus or Cytomegalovirus following cessation of the causative drug, the reactivation of latent virus is implicated in the long term sequalae seen with DRESS (Picard et al., 2010; Pichler and Brüggen, 2023).

DRESS is known to be induced by at least 50 drugs, including dapsone and ß-lactam antibiotics, with drug-specific CD4^+^ and CD8^+^ T-cells being identified in the skin and circulation of those affected (Naisbitt et al., 2003). Symptoms tend to appear between 2 and 6 weeks following initial drug administration and often include: fever, haematological abnormalities, and abnormal liver function alongside diverse manifestations of skin rashes such as urticaria or maculopapular eruption (De et al., 2018b). A regiSCAR study of 117 probable or definite DRESS patients found that 100% of patients experienced skin involvement, with over 50% body surface area covered in 79% of patients (Kardaun et al., 2013). This study found that aromatic anti-convulsants, including carbamazepine, and the anti-gout drug allopurinol were the most common primary causal drugs.

#### 1.1.5 Stevens-Johnson syndrome / toxic epidermal necrolysis

SJS/TEN is a spectrum disorder considered to be one of the most severe immune-mediated ADRs (Hasegawa and Abe, 2020). The condition is characterised by skin and mucosal membrane blistering and epidermal necrosis leading to large areas of epidermal detachment. SJS and TEN exist on the same spectrum and can be differentiated by the total body surface area affected with SJS being < 10% and TEN being > 30% (Wang et al., 2018). Despite intensive intervention, mortality is high with a regiSCAR study of 460 SJS/TEN patients finding overall mortality rates of 23% at 6-week post-reaction and 34% at 1-year post-reaction (Sekula et al., 2013). Short and long-term sequalae is also well-recognised and includes respiratory complications and sepsis (White et al., 2018).

Cytotoxic immune cells are implicated in the pathogenesis of SJS/TEN with the secretion of mediators such as granulysin and Fas Ligand by CD8^+^ T-cells present in blister fluid, and circulating natural killer cells directing keratinocyte death via apoptosis (Harris et al., 2016). CD40L is also found to be present which may contribute to the induction of apoptosis through TNF-α release (Oakley and Krishnamurthy, 2023). Drugs are overwhelmingly seen to be the causative agents in SJS/TEN and are similar to those seen with other cutaneous ADRs, including antibiotics, anti-epileptics, and allopurinol being the most commonly associated with the precipitation of SJS/TEN.

### 1.2 Drug hypersensitivity risk factors

Risk factors related to each of the DHRs previously mentioned are poorly understood and naturally contribute to the high incidence of these reactions despite a common pool of culprit drugs. Understanding the variations in mechanism and effector cells associated with these reactions may arise from inter-individual differences and the potential risk factors related to the induction of DHRs need to be scrutinised.

#### 1.2.1 Drug and patient-related factors

A multitude of factors relating to both patient and drug characteristics have been suggested to increase risk of DHRs. Epidemiological data suggest that female sex and advanced age are associated with higher incidence of DHR, the latter of which being associated with slowing of the immune system. Drugs with larger molecular weights are also thought to be more likely to bind and activate immune receptors although is known that reactive drugs with smaller molecular weights are capable of activating T-cells following the formation of protein-adducts (Gomes and Kuyucu, 2017).

Concomitant infection also likely predisposes an individual to developing DHR. The danger hypothesis, discussed in detail later, suggests that drug signals alone are insufficient to stimulate an immune response and a secondary signal, derived from pathogen induced damage may additionally be required to induce a hypersensitivity reaction.

#### 1.2.2 Genetic factors

The most extensively studied risk factor for DHRs are human leukocyte antigen (HLA) alleles associations. HLA genes are the most polymorphic genetic elements in the human genome, responsible for encoding the major histocompatibility complex (MHC) molecules.

MHC molecules play an essential role in both innate and adaptive immunity by presenting peptide antigens to T-cells. Associations between specific HLA alleles and protection from or predisposition to disease states have been established, including risk of autoimmunity and drug hypersensitivity. Risk alleles can be linked to specific drugs like vancomycin induced DRESS with HLA-A*32:01 or specific ADRs such as the casual association between FDE and the HLA-B*22 family (Konvinse et al., 2019; McClatchy et al., 2022).

The strongest allele association observed for SJS/TEN is between HLA-B*15:02 and carbamazepine in the Han Chinese population with pre-treatment screening currently in use. However, it should be noted that this risk allele is only relevant to SJS/TEN and has not been associated with carbamazepine-induced MPE or DRESS (Alfirevic and Pirmohamed, 2010). Epidemiological genome wide association and *in silico* binding studies have found several other drugs to be associated with HLA risk alleles, such as dapsone and HLA-B*13:01, although the positive predictive value (PPV) of such associations have proven insufficient to rely upon for clinical decisions (Zhang et al., 2013). HLA risk allele associations typically have low PPV, possibly due to the requirement for a complimentary TCR clonotype able to initiate an immune response rather than solely the ability of the HLA to present the drug for T-cell scrutiny.

Despite extensive characterisation of DHR manifestations our understanding of the underlying pathology is still incomplete, especially where distinct pathologies have overlapping origin. The low number of hypersensitivity patients available to study per individual drug further complicates efforts to develop meaningful predictive tools for potential hypersensitivity. Fully characterising the basic mechanisms of drug-induced T-cell activation which occur for most if not all drugs is therefore an essential step in identifying the likelihood of drug hypersensitivity.

### 1.3 Drug hypersensitivity reaction mechanisms

**FIGURE 1:**
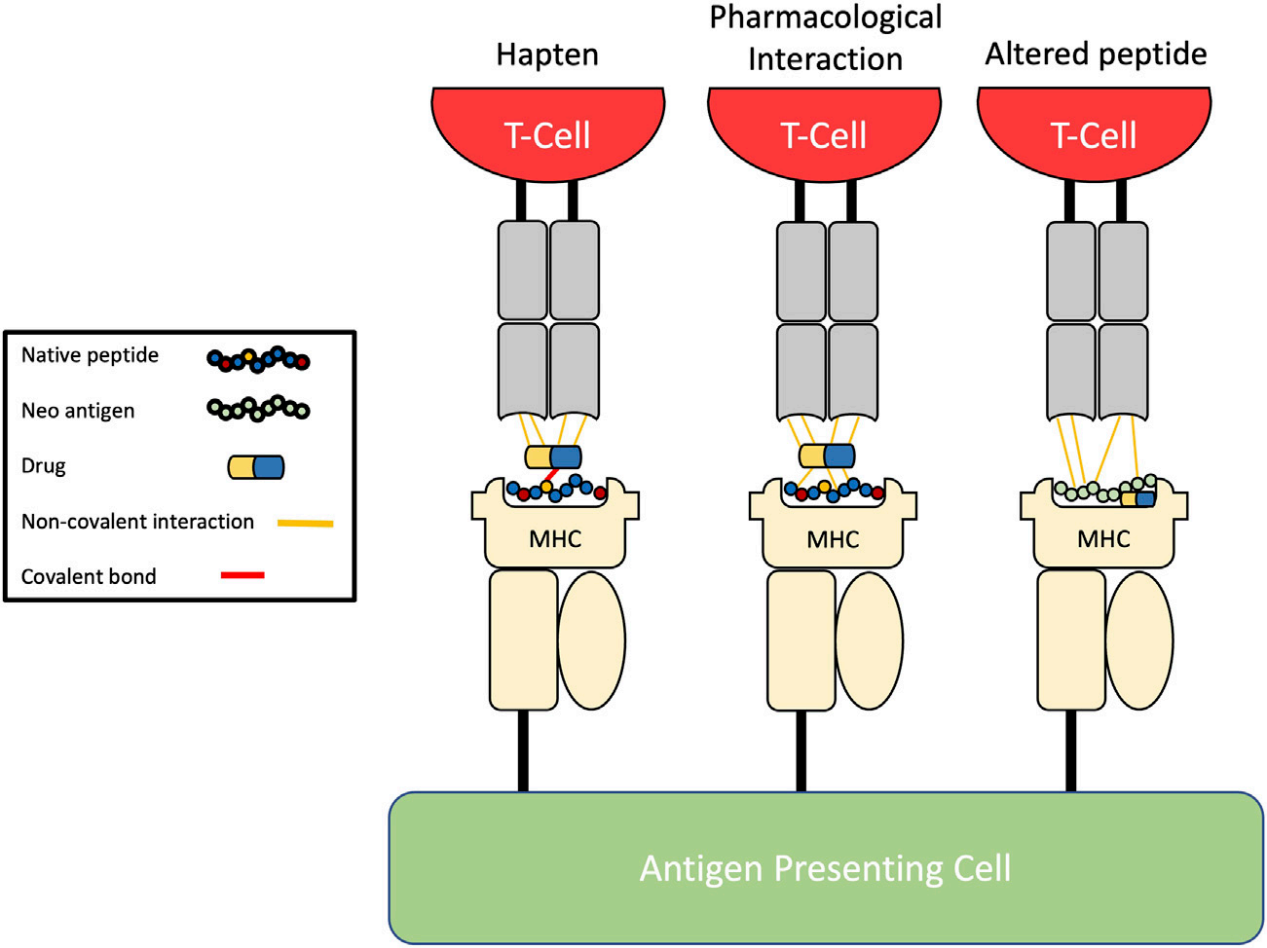
Pathways of drug-induced T-cell activation. The hapten model describes the covalent binding of drug to endogenous peptides before antigen processing leading to formation of neo-antigens which are then presented. The pharmacological-interaction (p-i) model describes the reversible non-covalent binding of drugs directly to the HLA molecule, antigen, or T-cell receptors without intracellular processing. The altered peptide repertoire model explains how a drug can alter the specificity of the peptide-binding groove through binding the F-pocket leading to presentation of unconventional self-peptides.

Three main pathways of T-cell activation by drugs have been proposed (Figure 1): the hapten model, the pharmacological interaction (p-i) model, and the altered peptide model.

#### 1.3.1 The hapten model

Landsteiner and Jacobs observed in 1935 that protein binding was necessary for small molecules to be able to induce skin sensitisation: a process which has come to be known as the hapten hypothesis (Basketter and Kimber, 2018). The hapten mechanism of antigen presentation involves chemically reactive compounds (i.e., drugs or their metabolites) covalently binding self-proteins resulting in the formation of a complex able to act as a neo-antigen in order to elicit any of the specific immune responses listed in Table 1.

A number of drugs frequently associated with skin-based hypersensitivity reactions are known to cause hapten modification, for example, the binding of penicillin drugs such as amoxicillin or flucloxacillin and their derivatives to serum albumin before antigen processing and presentation (White et al., 2014). One reason hapten-based reactions may be frequently implicated in DHRs is the inherent delay associated with drug-modified endogenous proteins moving through the stages of intracellular antigen processing before MHC loading and presentation (White et al., 2014). The penicillin family is frequently associated with cutaneous adverse reactions, particularly MPE, which has led to the pathogenesis being extensively studied (Goh et al., 2021). The extraction of penicillin modified human serum albumin from patients has confirmed the ability of penicillin type drugs to bind peptides and since then several peptides targeted by various ß-lactams have been identified and their immunogenicity interrogated.

#### 1.3.2 The pharmacological-interaction concept

Notwithstanding the early proposal of hapten formation as a mechanism of drug hypersensitivity, several non-reactive drugs, or diagnostic agents, such as radiocontrast media, have been known to induce adverse immune reactions in a manner non-conformant with the hapten model. In the early 2000s, Pichler et al. proposed the pharmacological interaction (p-i) concept as a manner of drug-induced immune activation without the need for antigen processing. Contrary to the hapten hypothesis, p-i reactions can rapidly manifest as this model postulates that drugs can non-covalently bind immune receptor proteins such as MHC or TCR and activate a response without the need for neo-antigen formation (Pichler, 2019). *In vitro* studies have provided substantial evidence for the model with this mechanism now being linked to the immunogenic properties of parent drugs such as carbamazepine and allopurinol (Pichler, 2019).

Certain T-cell mediated skin reactions, such as those seen in response to radiocontrast media, can occur rapidly and without prior sensitisation to the causative agent. While primary immune responses typically take days to manifest, the p-i concept may present the opportunity to bypass the innate immune system and instead activate a memory T-cell response. Although previous drug exposure is not necessarily seen in these reactions, the drug in question may be cross-reactive with specific memory T-cells which have a lower threshold of reactivity and generate a more rapid response than naïve T-cells as described in subsequent sections (Pichler, 2008). Certainly, this model is considered to bridge the gap between pharmacology and immunology with the binding of immune receptor proteins often considered to be an off-target effect of the drug. Although naturally the elicitation of a reaction is dependent on the affinity and orientation of drug binding to immune factors.

#### 1.3.3 The altered peptide repertoire

The final hypothesis of drug mediated T-cell activation is that of the altered peptide repertoire which was formulated following investigation of HLA-B*57:01 restricted DHRs to abacavir, an antiretroviral, which is to-date the only drug known to induce T-cell activation via this mechanism. 5%–8% of HIV patients taking abacavir experience a systemic hypersensitivity syndrome with multi-organ involvement such as skin rash and respiratory complications with the reaction even proving fatal in rare cases (Roen et al., 2018).

Distinct from the hapten and p-i models, this mechanism demonstrates that abacavir is capable of binding deep within the binding groove of HLA-B*57:01, specifically the F-pocket, thereby altering its specificity and subsequently the repertoire of antigen presented. The so-far unique altered peptide repertoire model explains what the hapten and p-i models cannot. Firstly, the hapten model would require abacavir to only modify HLA-B*57:01 specific self-peptides which is unlikely given the overlap in peptide repertoire between HLA molecules and the considerable similarity of the binding motif to abacavir insensitive HLA-B*58:01. Indeed, the similarities between abacavir sensitive HLA-B*57:01 and abacavir insensitive HLA-B*57:03 also make the p-i model improbable as the amino acid residue differences which distinguish between the two molecules is located deep in the binding groove and are unlikely to mediate TCR recognition (Ostrov et al., 2012). Despite this, it could be argued that the altered peptide repertoire model is merely a branch of the p-i model due to the similarities in mode of antigen binding.

Broadly, drugs can induce a diverse array of adverse immune reactions through distinct activation and effector pathways, particularly in the skin but to delineate the underpinning factors which polarise DHRs to cutaneous tissue, or any other target organ, will require focused investigations on what makes such tissues unique.

## 2 Why the skin?

The skin represents the most common target of immune-mediated adverse drug reactions (ADR) seen in the clinic. Clinical presentation of cutaneous ADRs is diverse, and ranges from localised minor self-limiting eruptions to severe, multi-organ, life-threatening pathologies. A significant question remains as to why this broad spectrum of drug-induced disease so frequently manifests in the skin; several key factors play a role in polarising reactions towards cutaneous tissue and more severe manifestations (Box 1). Furthermore, the mechanism behind the ability of a drug to drive diverse clinical manifestations between patients remains unsolved, as in the case of amoxicillin which can independently elicit MPE, AGEP and drug-induced liver injury (DILI).

Box 1 **Why drug exposure is frequently associated with cutaneous reactions.**

• Cutaneous reactions are readily visible so patients can self-report.
• Metabolism of drugs can occur in the skin, meaning drug antigen is present in the dermis and epidermis at high concentration.
• The skin is subject to continual assault from external factors, which induce danger signalling and favor an environment of elicitation.
• Immunosurveillance in the skin comprises of three distinct tiers which encompass the priming of naïve and activation of tissue resident memory T-cells.
• Virus specific skin resident memory T-cells can express pan-specific TCRs and mount reactions to drug antigen present in the skin in a cross-reactive manner.

Reports show that up to 1 in 100 clinical trial participants develop skin eruptions during the course of enrolment in their respective studies, demonstrating the high frequency of cutaneous reactions (Roujeau, 2005). This rate of disease is largely attributable to MPE, which accounts for over 90% of cutaneous manifestations seen in the clinic (Hunziker et al., 1997). One explanation for the high prevalence of skin involvement may be that such pathologies are easily identifiable. Patients are more likely to self-report cutaneous complaints to their healthcare provider without the need for invasive tests. In contrast, liver and acute kidney injury are likely to go unnoticed without active monitoring of transaminase elevation and serum creatinine respectively, upon initiation of a new drug regimen. Similarly, cutaneous drug hypersensitivity presentations are often the first symptom of a much wider systemic reaction. This may suggest that cessation of drug treatment at the first sign of cutaneous involvement would halt the progression of a so-far mild reaction to a more serious pathology such as DRESS.

While clinical factors such as these may contribute to the high prevalence of skin involvement compared to other organs, it is unlikely to be solely responsible for the heightened prevalence of cutaneous ADRs. The question of why drug exposure is most commonly associated with cutaneous disease is difficult to discern and is likely to derive from a complex interplay of factors between the resident immune system of the skin and the pharmacokinetic (PK) variables of a given drug.

### 2.1 Pharmacokinetics (metabolism)

PK characteristics of a given drug will play a significant role in determining the precipitation of an ADR and its severity. Route of administration, extent of distribution, metabolism and ultimately excretion of a drug all contribute to the amount of drug derived antigen present throughout the body. However, little research has been undertaken to quantify the location and amount of drug antigen generated, with virtually no studies looking at skin resident bioactivation of drugs *in vivo*.

The skin is a metabolically competent organ, and although not studied extensively, it has been shown to express a range of phase I and phase II enzymes including cytochrome P450s (CYPs), sulfotransferases, glutathione s-transferases and glucuronosyltransferases in all the layers of the skin (Gibbs et al., 2007). Throughout the last 20 years *in vitro* studies utilising *ex vivo* skin biopsies and reconstructed human skin have explored the metabolic potential of cutaneous tissue. Reilly et al. demonstrated in the early 2000s that epidermal keratinocytes are capable of oxidising well-known sulfone antimicrobials, dapsone and sulfamethoxazole, into their reactive hydroxylamine metabolites, both of which are associated with SJS and DRESS (Reilly et al., 2000; Pratoomwun et al., 2021). These studies demonstrate that cutaneous tissue possesses the innate capacity to metabolise drug but does not address how drugs may be able pass from the circulation into close proximity with tissue resident enzymes and subsequently the effectors of an immune response.

Significant work has been undertaken to investigate the mechanisms by which drugs and allergens enter the skin, although studies are generally skewed towards those from the external environment which cause allergic contact dermatitis (ACD). While there are clear similarities between ACD and drug hypersensitivity to systemic treatment, the former has the benefit of direct allergen exposure at the site of reaction. Here, allergens pass through the hard outer layer of skin (the stratum corneum) and into the epidermis, where they can be bioactivated or interact unchanged with specialised antigen presenting cells (APCs) to initially prime naïve T-cells and then elicit a full T-cell mediated reaction upon subsequent exposure (Mustafa et al., 2018).

Contrastingly, drug hypersensitivity to systemic treatment is often a single step process by which T-cells can be activated and clonally expand from initial exposure, highlighting a clear disparity between the two pathologies. Additionally, drugs (or drug antigen) which are present in systemic circulation requires inverse trafficking compared to those seen in ACD, i.e., out of systemic circulation, through the dermis and ‘localisation’ to the epidermis where tissue damage occurs. Existing studies describe in detail the localisation and metabolism of topically applied allergens, such as aromatic compounds which can undergo epoxidation in the skin, but it is still unknown how and to what extent this applies to systemically administered compounds (Giles et al., 1997; Gibbs et al., 2007).

It has been previously demonstrated that systemically administered drugs can pass out of the circulation into the skin, both unchanged and following first pass metabolism. *In vivo* studies conducted in murine models show that just under 20% of a systemically administered anticonvulsant, lamotrigine, localises to cutaneous tissue (Maggs et al., 2000). Clearly, drugs are able to distribute to the skin either as parent or metabolite, as is required for the excretory function of the skin.

Membrane transport proteins have a role in controlling local concentrations of drug in the skin, both intracellularly and in interstitial fluid. ATP-binding cassette (ABC) and solute carrier (SLC) transporters have been observed in the skin, at varying levels. ABCC1, a known drug transporter, is known to be expressed in the skin, in addition to ABCF1, a transporter critically involved in the movement of cytokines and chemokines between cells which is essential for an immune response (Takenaka et al., 2013). There is some evidence to suggest that the differential expression of transporters in the skin between individuals will influence their susceptibility to disease as in the case of melanoma patients who have lower cutaneous expression of ABC transporters compared to healthy individuals, meaning drugs such as gemcitabine accumulate in the skin and causes SJS (Sommers et al., 2003).

While transporter expression will affect the accumulation of drugs in the skin, there is large inter-individual variability with regard to the population of transporters expressed (Fujiwara et al., 2014). There is little data available to interrogate trends between drugs which elicit skin-specific reactions and any individual class of transporter, there remains a substantial area of research yet to be undertaken.

Similarly, there is little *in vivo* evidence of cutaneous phase I bioactivation of systemically derived drug compounds (Roychowdhury and Svensson, 2005). Phase I enzymes are expressed at far lower levels than phase II enzymes in the skin with sulfotransferases and acetyltransferases representing the enzyme classes with the highest activity in cutaneous tissue (Sharma et al., 2019). Most drugs will enter the skin altered by first pass metabolism and the function of the skin as an excretory organ permits the elimination of these phase I metabolites through sweat via conjugation to large groups hydrophilic groups, (Zhou et al., 2012). Compounds destined to be excreted through sweat and sebum in the skin will however pass only into the lower dermis where they are taken up by sweat or sebaceous glands (Folk and Semken, 1991).

Most reactive metabolites are generated through oxidation, largely by members the CYP superfamily of enzymes. Langerhans cells and keratinocytes in the epidermis have been observed to express metabolising systems containing CYP families 1 to 3; pointing to the viable epidermis as the main location where biotransformation occurs in the skin (Pyo and Maibach, 2019; Sharma et al., 2019). Furthermore, the epidermis is the main location where drug antigen interacts with professional APCs to subsequently be presented to naïve T-cells in the skin draining lymph nodes. It could be argued that the frequency of cutaneous manifestations is high because the critical step in the pathogenesis of ADRs occurs in the epidermis, where drugs are bioactivated into chemically reactive species. This in turn leads to formation of neoantigens via haptenation of largely keratinocyte derived proteins (Svensson, 2009).

However, there are limitations to this argument. Primarily, orally administered drugs will disperse through the liver prior to entering systemic circulation, such that a large proportion of drug is changed via first pass metabolism. Numerous organs, including the liver, lungs, and kidneys, all have metabolic capacity similar to that, if not greater than the skin such that similar bioactivation events will occur in these tissues. Chemically reactive metabolites are generated globally, and protein reactivity is a common occurrence, but contrastingly, immune-mediated ADRs are not observed in these organs as frequently. Pichler et al. noted that most patients administered the antimicrobial amoxicillin will ubiquitously generate an abundance of new neoantigens throughout the body, but the majority of patients on this medication will not develop a T-cell mediated skin reaction suggesting a critical factor in the skin is missing elsewhere, such as the tissue resident immune environment (Pichler et al., 2002).

Studies conducted on *ex vivo* skin samples identified that of over 85 metabolising enzymes present at the mRNA level, only 26 were translated and expressed as protein (Kazem et al., 2019). Whilst the skin, among several other organs, has the necessary machinery to metabolise xenobiotics and there is substantial evidence that their activity can have a distinct effect on local drug concentrations, the contribution of cutaneous drug metabolism, compared to that of the liver and other organs is relatively small, with phase I metabolic pathways having around 300 times less activity (van Eijl et al., 2012). Drug metabolism can be enzyme specific and therefore differences in tissue expression of metabolising enzymes such as CYPs will have an impact on the generation of reactive metabolites in each tissue. The majority of drugs are metabolized by CYP families 2 and 3, which are present in both the skin and liver (Zhao et al., 2021).

Whilst studies have shown that drug protein adducts formed within the skin have been sufficient to activate T-cells (Elsheikh et al., 2011), drug antigen will be produced throughout the body at varying levels, the majority of which emerging from hepatic metabolism. Reactive metabolites formed in the liver have been shown to be present in circulation, which makes the highly vascularised skin particularly vulnerable to increased exposure of a given drug or drug antigen.

It remains unclear the extent to which local generation of drug antigen contributes towards the initiation of a skin-based reaction; however the determining factors in polarising reactions towards the skin is likely more complex and is compounded by local variables such as transporter protein expression, which impact accumulation of metabolites, and the state of the local immune environment.

### 2.2 Immune environment

Reactivity remains the natural status of the immune environment in the skin, where the innate and adaptive immune system constantly surveil, seeking foreign entities which may cause harm to the body. This constant state of heightened alert is central in maintaining the integrity of the physical barrier function of the skin and a healthy interface with the external environment (Gibbs et al., 2007). The protection afforded by the skin means it is continually subject to an onslaught of danger signals which drive inflammation in response to allergens and pathogens, among other environmental insults.

Damage-associated or pathogen-associated molecular patterns (DAMPs and PAMPs) are the most common form of danger signal and activate pattern recognition receptors. For example, TLR1-5 which are expressed on the surface of professional APCs like immature dendritic cells (DCs) (Flacher et al., 2006; Schreibelt et al., 2010). DCs and their skin resident counterpart Langerhans cells are sentinels of the immune system, responsible for taking up and presenting antigen to T-cells in the context of MHC. Activation of a TLR promotes DC maturation and subsequent migration to skin draining lymph nodes where they upregulate numerous cell surface markers including co-stimulatory molecules such as CD80 and CD86 while downregulating co-inhibitory molecules such as PD-L1 (Esser et al., 2023).

DAMPs and PAMPS can be generated via various means including cellular stress, mitochondrial injury, the unfolded protein response (UPR) or inflammasome activation. These processes can result from UV skin damage, inflammatory and autoimmune diseases such as arthritis, eczema and psoriasis, or viral and bacterial infection (Weston and Uetrecht, 2014; Man and Kanneganti, 2015). DAMPs and PAMPs are traditionally proteins, polysaccharides, or nucleic acids such as heat shock proteins, virus derived ssRNA or bacterial cell wall components (Tang et al., 2012). Antimicrobials and antivirals are some of the most common therapeutics to cause cutaneous hypersensitivity reactions. Patients are generally on these medications to treat acute or chronic infection, as is the case with ß-lactams which are indicated for a wide range of diseases, from minor ear nose throat infections to recurrent pneumonia in cystic fibrosis patients (Lyczak et al., 2002). These co-morbid infections produce substantial PAMPs which can, in turn, cause bystander activation of T-cells independent of TCR engagement (Whiteside et al., 2018).

The danger hypothesis was first proposed in 1994 by Polly Matzinger as a substitute for the non-self-hypothesis, suggesting that in the absence of tissue damage and inflammation and therefore danger signals, an immune response will not be mounted. Where engagement of a TCR and an antigen loaded MHC molecule (Signal 1) occurs in healthy tissue, the natural immune response will be tolerance. However, in an environment that is congested with danger signals such as the skin, these danger signals act as “Signal 2,” an adjuvant which upregulates the expression of co-stimulatory receptors such as B7 (CD80 and CD86) on DCs. These factors then engage with CD28 on T-cells to tip the balance of the immune response away from tolerance into a reactive state (Matzinger, 1994; Matzinger, 1998).

Drugs and their metabolites under certain circumstances may be capable of producing DAMPs through production of reactive oxygen species which oxidise proteins, creating neo-antigens and applying substantial ER stress through induction of the UPR, an important driver of inflammation and immune activation (Weston and Uetrecht, 2014; Hetz et al., 2020). Co-stimulatory and co-inhibitory molecules driven by the presence or absence of danger signals have a significant role in regulating whether recognition of drug antigen by a TCR leads to activation and subsequently tissue damage. Danger signalling is key for the full activation of an immune response, particularly in the context of drug allergy. The constitutive presence of danger signals within the skin is sufficient to lower the background threshold required to elicit an immune reaction in response to drug antigen which would otherwise be tolerated (Uetrecht, 2007). It follows that constituent danger signalling within the skin likely creates an environment which is integral to predisposing cutaneous tissue to a higher frequency of adverse drug reaction than other organs.

### 2.3 Immune surveillance

Immune surveillance within the skin is complex and multi-faceted, with immune cells continually circulating into and out of cutaneous tissue via blood vessels and the lymphatic system. While circulating immune cells play a key role in surveillance, there are a number of specialised immune components which reside in the dermis and epidermis. For example, macrophages, keratinocytes, Langerhans cells, and DCs, are all involved in the primary, secondary and tertiary surveillance which contribute to a strictly regulated and comprehensive immune environment (Kupper and Fuhlbrigge, 2004). Each level of surveillance likely has a part to play in the initiation of cutaneous hypersensitivity reaction, the severity and progression.

Primary surveillance occurs where DCs, antigen and naïve T-cells are brought together and interact within a secondary lymphoid organ (SLO). Langerhans cells and dermal DCs detect DAMPs and PAMPs in the local environment causing the uptake and processing of antigen onto MHC Class II molecules. Subsequently, these cells mature and migrate to the local draining lymph nodes where they present the antigen to naïve (or central memory) T-cells promoting their activation and differentiation into effector subsets in an antigen-dependent manner, which then distribute to the site of primary stimulation (Banchereau and Steinman, 1998). Primary surveillance is integral to delayed-type drug induced injury where it reflects the naïve priming model and the latency period often seen relates to the time taken for naïve T-cells to be sensitised, migrate, and elicit a reaction.

Secondary surveillance describes the process by which effector memory T-cells that express skin homing molecules such as cutaneous lymphocyte antigen (CLA) and CCR4 continually traffic into and out of the skin, searching for antigen. This process readily occurs in healthy skin but is increased during inflammation where the surrounding tissue and vasculature upregulate cell adhesion molecules and skin-homing chemokines to recruit memory T-cells in an antigen-independent manner. This increases the likelihood that an effector memory cell with an appropriate TCR interacts with antigen in the local environment without the need for an antigen to be presented in a SLO (Kunkel et al., 2002; Kupper and Fuhlbrigge, 2004). Secondary surveillance likely accounts for a large proportion of immediate or semi-delayed drug reactions, where drug derived epitope that is present in the skin is recognised by skin homing memory T-cells generated from a previous encounter with (a) the specific drug or metabolite, (b) a structurally similar drug or (c) from a pathogen derived epitope which has a similar sequence.

Tertiary surveillance specifically encompasses the activity of CD62L^+ve^ and CCR7^+ve^ central memory T-cells. Central memory cells traffic through lymph nodes across the body, migrating to areas distinct from that which they originated and enacting rapid immune responses at both healthy and injured sites, different from their tissue resident counterparts (Sallusto et al., 1999; Campbell et al., 2001). Tertiary surveillance likely plays a role in the progression of cutaneous adverse reactions into systemic or more widespread disease like the progression of MPE to DRESS or SJS/TEN.

SCARs are often distinguishable from other delayed-type hypersensitivity reactions such as those which manifest in the liver in terms of cellular phenotype and the effector molecules responsible for the clinical manifestations. Multiple studies have identified CLA and lymphocyte function-associated antigen (LFA-1) expression on the surface of drug-specific T-cells in hypersensitivity patients, which suggests that a given drug can selectively induce the expansion of tissue specific, in this case CLA^+ve^, T-cells either from naïve or effector memory pools (Naisbitt et al., 2003; Gibson et al., 2023). Migration to specific tissue is efficiently regulated by cell surface markers, such as CLA which is expressed on up to 30% of circulating memory T-cells. CLA in particular is responsible for tethering and rolling of T-cells on E-selectin, which is constitutively expressed on dermal vasculature (Picker et al., 1990). Similarly, the expression of CCR4, CCR10, CCL17, and CCL27, is drastically elevated, the last of which is produced by keratinocytes plays a significant role in attracting memory T-cells to the skin (Kupper and Fuhlbrigge, 2004).

Little is known concerning the factors which promote the expression of skin-homing receptors on activated T-cells, but it is likely to result from the latent environment within the local lymphatic organ which the naïve T-cell was initially activated. This is demonstrated by studies which show that rotavirus specific T-cells activated in Peyer’s Patches express the gut-homing receptor α_4_β_7_ and localise to the gut mucosa. Similarly, herpes simplex virus II specific T-cells activated in skin draining lymph nodes express CLA (Rott et al., 1997; Koelle et al., 2002). This evidence suggests that drug reactive T-cells, which are responsible for the skin injury seen in SCARs, derive from naïve or memory T-cells initially primed to an antigen that is expressed on keratinocytes within a skin draining lymph node. Altogether this indicates that drug antigen must be capable of moving from circulation, through each layer of the skin and be taken up by the target T-cell in order to induce a DHR.

### 2.4 Memory T-cells

Research into memory T-cells and their role within SCARs is a rapidly growing area of interest. Memory T-cells act like a repository of antigenic determinants which a person has been exposed to and these cells gradually accumulate throughout a person’s lifetime. Up to 35% of the T-lymphocyte population in an individual consists of memory phenotype cells before the age of 30 (Cossarizza et al., 1996). This pool of memory T-cells forms a wide-reaching component of the immune system. Following activation of a naïve T-cell through antigen engagement, clonal expansion occurs where T-cells differentiate into either effector and memory populations, the latter of which is composed of circulating and tissue resident groups.

Central memory T-cells (TCMs) express high levels of CD62L and CCR7 which retains them in secondary lymphoid tissues. TCMs have a high proliferative capacity upon activation but are slow to produce effector cells where needed. Conversely, effector memory T-cells (TEMs) lack these cell surface molecules, thus permitting their circulation throughout the body and enabling a rapid effector function wherever required. TCM and TEM subsets are integral to tertiary surveillance and will respond to re-challenge of specific antigen anywhere it is present within the body, however a proportion of TEMs (both CD4^+^ and CD8^+^) will switch to a tissue resident memory T-cell (TRM) phenotype which home to and are retained in a specific tissue (Gray and Farber, 2022).

Skin resident memory T-cells express various cell surface markers (CD69, CD103 and CLA) which blocks their egress out of the dermis and back into circulation (Schunkert et al., 2021). TRMs undergo metabolic changes which lower turnover rates, thus causing the gradual accumulation of TRMs at mucosal sites (Gray and Farber, 2022). Memory lymphocytes generally only establish residency following antigen encounter as per the naïve priming model. This means that TRMs tend to be specific to antigen that is found in the tissue of residency.

Presently, the exact location and mechanism by which T-cells are stimulated in the skin by drug antigen remains a significant unanswered question. Naturally, drug antigen must be distributed to the location of T-cell lodgment, which suggest TRM T-cells play a crucial role to disease pathogenesis and the restriction of T-cell mediated immune responses to determined tissues (Osman et al., 2023). Studies have demonstrated the sole importance of TRMs to the pathogenesis of more severe forms of SCARs like SJS/TEN. For example, cancer patients which have been ablated of any circulating T-cells, are still able to mount a T-cell mediated delayed hypersensitivity reaction derived from CD45RO^+^/CD69^+^ TCMs (Iriki et al., 2014). This evidence warrants the further investigation into the specific role which TCMs play in SCARs and how they may be further implicated in the progression of such disease throughout and beyond the skin.

Further implicating TRMs in disease pathogenesis, studies have observed that the TCR sequence from fixed drug eruption patient T-cells derive from local cytotoxic TRMs rather than the central memory pool (Strobl and Haniffa, 2023). Additionally, TCR repertoire analysis in TEN patients revealed massive clonal expansion of CD8^+^ lymphocytes which derive from a TEM phenotype, providing further supporting evidence of their involvement (Villani et al., 2021). The longevity of TRMs in the skin varies and some will undergo migration during their lifetime to seed distinct organs from the tissue of origin with between 15% and 30% of circulating T-cells being ex-tissue resident (Wijeyesinghe et al., 2021). CD4^+^ TRMs are capable of downregulating CD69 to exit the skin and migrate to secondary sites where they can resume TRM phenotype This process, known as the Koebner effect, may explain how cutaneous hypersensitivity reactions can spread across the entire body, migrating and seeding healthy tissue where they can be activated by local drug antigen exposure (Klicznik et al., 2019).

### 2.5 Heterologous immunity

**FIGURE 2:**
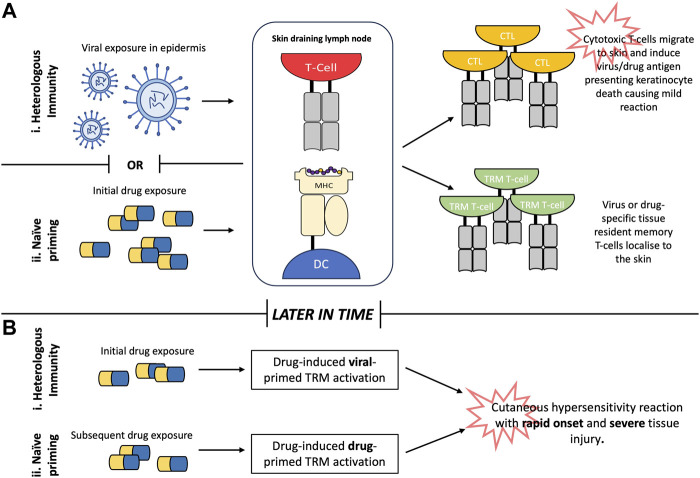
Generation of drug-reactive T-cells in the skin. **(A)**. Heterologous immunity describes how individuals retain tissue resident T-cells specific to viral epitopes within the skin following viral infection in early life. Subsequent exposure to drug antigens with sufficient homology to the viral epitope facilitates cross-reactivity with viral-primed tissue-resident memory (TRM) T-cells causing rapid onset of T-cell mediated tissue injury. **(B)**. Conversely, naïve priming describes how initial exposure to drug antigen primes naïve T-cells to generate drug specific TRM T-cells which are rapidly reactivated upon future re-exposure to the drug in question. Abbreviations: MHC (major histocompatibility complex), DC (dendritic cell), CTL (cytotoxic T-lymphocyte), TRM (tissue resident memory).

Heterologous or “cross reactive” immunity is a function in T-cell immunology commonly viewed in relation to vaccination and describes how protection against one pathogen can confer protection against another via recognition of similar antigenic determinants. Heterologous immunity in the context of cutaneous hypersensitivity has gained traction particularly in explaining the tissue specificity of SCARs and how drug naïve patients can mount an immune response upon first exposure to a given drug. This is the case for abacavir hypersensitivity syndrome, where specific T-cells have been identified in drug naïve donors (Adler et al., 2017; Gibson et al., 2023). Cross-reactive immunity is a departure from the traditional naïve priming model commonly associated with the induction of drug hypersensitivity reactions and does not require a “sensitisation step” whereby the drug is presented to a naïve T-cell in a skin draining lymph node. Instead rapid T-cell responses can be mounted to a drug antigen that shares sequence similarity to viral epitopes (Figure 2).

There is already substantial knowledge on cross reactive immunity as it relates to viral disease and the induction of auto-immune responses where TRMs specific to viral epitopes recognise self-antigens and provide the tissue specificity for the clinical phenotypes seen with cutaneous reactions. Epitopes presented in the context of MHC only make 2-3 contacts with the TCR during antigen recognition, meaning homology between viral and drug-derived antigen may only relate to a small number of residues increasing the likelihood of cross-reactivity. Polyspecific T-cell receptors are capable of recognising multiple distinct peptides, including several derived from allogenic targets (Welsh and Selin, 2002).

The memory T-cell pool of a patient grows with each successive infection and the capacity for a patient to cross-react heavily depends on their history of prior infection. Studies have identified that CMV specific T-cells account for a significant proportion of the total memory T-cell repertoire (White et al., 2015). Equally cytotoxic T-cells mediating the cutaneous symptoms of DRESS have been shown as Epstein-Barr virus (EBV) reactive *in vitro*, demonstrating the capability of polyspecific T-cells to enact drug reactivity (Picard et al., 2010). Tissue specific expression of self-peptides significantly influences the propensity for tissue specific pathologies. Self or drug derived peptides need to be present in proximity to virus specific TRMs, as with the case of EBV specific T-cell responses that are observed as cross reactive to allogenic targets limited to the skin and mucous membranes; where a peptide similar to EBV nuclear antigen 3a is preferentially presented on the surface of keratinocytes and endothelial cells (D'Orsogna et al., 2011). Heterologous immunity accounts for the durable reactivity that is observed in patients recovering from cutaneous hypersensitivity reactions, where ordinarily a waning of response would be expected following long-term cessation of treatment however persistent pathogen encounters maintains these populations of polyspecific TRMs (Welsh and Selin, 2002).

Furthermore, memory T-cells have a much lower threshold for activation, requiring up to fifty times less antigen than naïve T-cells for stimulation (Curtsinger et al., 1998). Therefore, immunodominant peptides are not the only antigenic determinants which can break T-cell tolerance with cross reactive responses often being mounted to peptides that do not share substantial homology to that the T-cell was originally primed. This observed promiscuity may account for the increased frequency of adverse drug reaction specifically in the skin, derived from cutaneous TRMs (White et al., 2015).

## 3 Discussion

Tissue specificity of T-cell mediated hypersensitivity reactions remains a complex, diverse, and under-represented field of study which encompasses multiple factors, largely relating to the tissue resident immune environment. There is unlikely to be a sole reason why drug exposure is frequently associated with cutaneous manifestations of T-cell mediated hypersensitivity but instead these reactions likely derive from the circuitous interplay between each of the previously mentioned variables.

Local drug metabolism naturally has an impact on the level of protein modification and neo-antigen formation which occurs within the skin. Although the metabolic capacity of cutaneous tissue does not exceed that seen in the visceral organs such as the lungs, liver or kidneys and therefore does not provide sufficient explanation for the high frequency of reaction which concentrate on the skin. Whilst drug metabolism may be a contributing factor to the development of cutaneous hypersensitivity reactions, the unique immune environment is fundamental to the function of the skin and must be considered.

The inflammatory nature of the skin and its complex immune environment does somewhat demonstrate how reactivity to drug antigen may be favored in cutaneous tissue while tolerance is the default position elsewhere. Constitutive danger signalling, coupled with three tiers of immune surveillance capability means the immune system in the skin is perpetually in a state of alert thereby predisposing the skin to immune activation. Drug antigen localisation and heterologous immunity also provide insight into how T-cell mediated responses may be directed towards the skin as antigen presentation is restricted to specific tissue. The idea that “no one is naïve”, a phrase used by Welsh et al., is an emerging concept in drug hypersensitivity. It describes a theory by which antigen specific T-cells may possess a polyspecific TCR capable of cross reacting to an antigen that has not previously been encountered based on memory of structurally similar antigens (Welsh and Selin, 2002). Correspondingly, there is renewed interest and mounting evidence suggesting a central role in the pathogenesis of cutaneous disease for memory T-cells—specifically TRMs able to mediate the localised reactions discussed in this review.

However, it is not adequately understood what polarises cutaneous reactions towards more severe manifestations such as SJS/TEN over DRESS or MPE and while the strength of response may relate to the number and phenotype of specific T-cell precursors present in the skin, little work has been undertaken on identifying the differences (Villani et al., 2021). Interestingly, the elimination half-life of a drug has also been associated with the severity and prognosis of SJS/TEN. Whilst some literature proposes that drugs with long half-lives are more likely to induce SJS/TEN, there is more convincing evidence to suggest that it is the severity and by association prognosis which is influenced by drug half-life (Oakley and Krishnamurthy, 2023). Disregarding the likelihood of toxicity induction, drugs with long half-lives are associated with poorer patient prognosis even in cases of early drug withdrawal when compared to drugs with short half-lives (Harr and French, 2010).

Antibiotics typically have short half-lives compared to anti-epileptics, however studies investigating SJS/TEN risk factors have repeatedly identified similar pooled risk with antibiotic associated SJS/TEN and anti-epileptic associated SJS/TEN (28% and 19% respectively). The more widespread use of antibiotics than anti-epileptics in the clinic should naturally be considered (Borrelli et al., 2018; Lee et al., 2023).

Similarly, a single drug can elicit distinct pathologies which extend into other target organs, such as DILI. It is yet to be elucidated why a given drug may cause MPE in one patient but DILI in another, such as Amoxicillin. It is also unclear why untreated MPE would progress to a more serious pathology such as DILI. Further research is needed at the single cell level to investigate differences in immune populations and unravel why a single antigen can elicit several distinct diseases.

Additional research which addresses not only skin-specific factors, but also intra/inter-individual variability is required to disentangle the skewed frequency of cutaneous involvement. This may include investigations into the influence of individual microbiota on the cutaneous immune environment and tissue specific epigenetic differences in key components of antigen processing such as TAP and ERAAP which in turn influence peptide cleavage and loading (Sun et al., 2023). Further pharmacogenomics approaches investigating the relationship between drug-associated HLA alleles and TCR clonotype may provide foundations for more robust patient screening and address the balance required to prevent hypersensitivity reactions whilst ensuring that patients receive optimal drug treatment.

**FIGURE 3:**
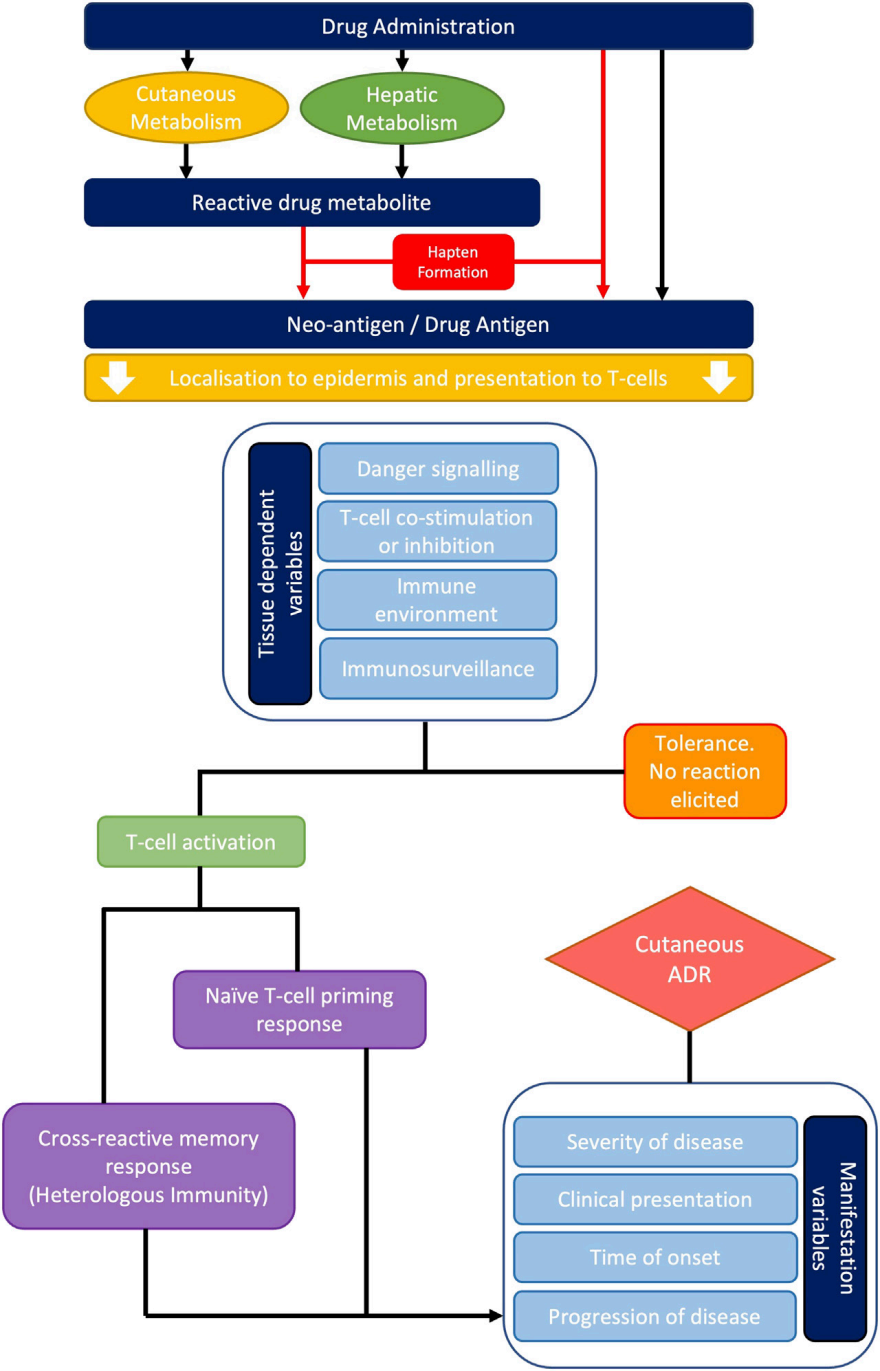
Overview of the typical pathogenesis of cutaneous adverse drug reactions. Systemically administered drugs undergo metabolism and/or endogenous processing resulting in hapten formation. Drug antigens can then be presented to naïve or cross-reactive T-cells in the context of MHC molecules. Multiple variables including danger signalling, co-stimulation, co-inhibition, and the local T-cell precursor population will either promote T-cell activation or tolerance. Where a reaction is mounted, the severity of disease, clinical presentation, time of onset and progression of disease will vary dependent upon the principal T-cell phenotype present.

Overall, it is likely that the frequent involvement of cutaneous tissue in T-cell mediated ADRs derives from the inherent nature of the immune environment of the skin and multi-faceted compounding factors. These include danger signalling, active immune surveillance, memory responses and heterologous immunity which form a ‘perfect storm,’ for targeting the bodies largest and most exposed organ (Figure 3).

The authors refer the readers to the following review articles for further reading; Gibson et al., 2023 (PMID: 36740326) covering the immunopathology of SCAR. Pyo and Maibach 2019 (PMID:31357203) covering pharmacologically relevant skin metabolism. Schunkert et al., 2021 (PMID: 34497600) specifically covering skin resident memory T-cells and their role in DHRs. Welsh and Selin 2002 (PMID: 12093008) covering heterologous immunity in detail.

## Figures and Tables

**Table:** 

**ACD**	Allergic contact dermatitis
**ADR**	Adverse drug reaction
**AGEP**	Acute generalised exanthematous pustulosis
**APC**	Antigen presenting cells
**CLA**	Cutaneous lymphocyte antigen
**CMV**	Cytomegalovirus
**CYP**	Cytochrome P450s
**DAMP**	Damage-associated molecular patterns
**DC**	Dendritic cell
**DHR**	Drug hypersensitivity reaction
**DILI**	Drug induced liver injury
**DRESS**	Drug reaction with eosinophilia and systemic symptoms
**EBV**	Epstein-Barr virus
**ER**	Endoplasmic reticulum
**ERAAP**	Endoplasmic reticulum aminopeptidase associated with antigen processing
**FDE**	Fixed drug eruption
**HIV**	Human immunodeficiency virus
**HLA**	Human leukocyte antigen
**LFA**	Lymphocyte function-associated antigen
**MHC**	Major histocompatibility complex
**MPE**	Maculopapular exanthema
**NSAID**	Non-steroidal anti-inflammatory drug
**PAMP**	Pathogen-associated molecular patterns
**PD**	Pharmacodynamics
**PK**	Pharmacokinetics
**PPV**	Positive predictive value
**SCAR**	Severe cutaneous adverse reaction
**SJS**	Stevens-Johnson syndrome
**SLO**	Secondary lymphoid organ
**TCM**	Central memory T-cell
**TCR**	T-cell receptor
**TEM**	Effector memory T-cell
**TEN**	Toxic epidermal necrolysis
**TLR**	Toll-like receptor
**TNF**	Tumour necrosis factor
**TRM**	Tissue resident memory T-cell
**UPR**	Unfolded protein response
